# The Pros and Cons of Getting Engaged in an Online Social Community Embedded Within Digital Cognitive Behavioral Therapy for Insomnia: Survey Among Users

**DOI:** 10.2196/jmir.5654

**Published:** 2016-04-25

**Authors:** Neil S Coulson, Richard Smedley, Sophie Bostock, Simon D Kyle, Rosie Gollancz, Annemarie I Luik, Peter Hames, Colin A Espie

**Affiliations:** ^1^ Division of Rehabilitation & Ageing School of Medicine University of Nottingham Nottingham United Kingdom; ^2^ Big Health London United Kingdom; ^3^ University of Southampton Faculty of Medicine Southampton United Kingdom; ^4^ Sleep and Circadian Neuroscience Institute (SCNi) Nuffield Department of Clinical Neurosciences Oxford United Kingdom

**Keywords:** engagement, sleep, online community, discussion forum, insomnia, cognitive behavioral therapy

## Abstract

**Background:**

Sleepio is a proven digital sleep improvement program based on cognitive behavioral therapy techniques. Users have the option to join an online community that includes weekly expert discussions, peer-to-peer discussion forums, and personal message walls.

**Objective:**

The aim of this study was to conduct an online survey to (1) explore the reasons for deciding to engage with the Sleepio online community, (2) explore the potential benefits arising from engagement with the online community, and (3) identify and describe any problematic issues related to use of the online community.

**Methods:**

We developed an online survey and posted an invitation to the community discussion forum inviting users to participate. In addition, we sent an email invitation to 970 individuals who had previously or were currently working through the Sleepio program to participate in this study.

**Results:**

In total, 100 respondents (70/100, 70% female; mean age 51 years, range 26–82 years) completed the online survey. Most respondents had started Sleepio with chronic sleep problems (59/100, 59% up to 10 years; 35/100, 35% >10 years) and had actively engaged with the online community (85/100, 85%) had made a discussion or wall post). At the time of the survey, respondents had used Sleepio for a median of 12 weeks (range from 3 weeks to 2 years). We analyzed responses to the open-ended questions using thematic analysis. This analysis revealed 5 initial drivers for engagement: (1) the desire to connect with people facing similar issues, (2) seeking personalized advice, (3) curiosity, (4) being invited by other members, and (5) wanting to use all available sleep improvement tools. Advantages of engagement included access to continuous support, a reduced sense of isolation, being part of a nonjudgmental community, personalized advice, positive comparisons with others, encouragement to keep going, and altruism. We found 5 potential disadvantages: design and navigation issues, uncertain quality of user-generated content, negative comparisons with others, excessive time commitments, and data privacy concerns. Participants related their community experiences to engagement with the Sleepio program, with many stating it had supported their efforts to improve their sleep, as well as helping with adherence and commitment to the program. Despite some concerns, members regarded the Sleepio community as a valuable resource.

**Conclusions:**

Online communities may be a useful means through which to support long-term engagement with Web-based therapy for insomnia.

## Introduction

Epidemiological studies suggest that the prevalence of clinical insomnia disorder ranges from 10% to 12% worldwide [1,2], and problems are often long lasting [3]. Insomnia disorder is defined as a combination of difficulty initiating and maintaining sleep, but it also has a significant negative impact on daytime functioning [4]. A range of negative daytime consequences are typically linked to insomnia, including increased fatigue, diminished work productivity, lower quality of life, and lower relationship satisfaction, as well as increased incidence of poor health [5]. Furthermore, the development of mental and physical health problems has been linked to chronic insomnia [6,7].

While pharmacological treatments have an evidence base for improving insomnia, patients often report a preference for cognitive behavioral therapy (CBT) [8]. Indeed, the evidence for CBT as an effective treatment is persuasive [9-11]; however, access is limited due to resource and expertise constraints [12-15]. As a result, attention has turned to the Internet as a means to deliver treatment for chronic insomnia [16]. Furthermore, accumulating evidence in recent years suggests that such interventions can provide effective treatment for patients [17-19]. For example, one of the most rigorous studies of Web-based CBT used a randomized, placebo-controlled trial design to examine the impact of participation in the Sleepio intervention. The findings revealed that CBT delivered through a media-rich Web app with automated support and a community forum was effective in improving the sleep and associated daytime functioning of adults with insomnia disorder [19,20].

Sleepio is a subscription-based, fully automated digital sleep intervention that delivers CBT-based content to patients with chronic insomnia via the Web and mobile phones [21]. The course is delivered through 6 weekly sessions, facilitated by an animated character, “The Prof.” As each session begins, The Prof reviews progress, and examines the diary data submitted during the week, current sleep status and pattern, and progress made toward goals previously set. Automated algorithms tailor CBT content, incorporating behavioral (eg, stimulus control), cognitive (eg, thought restructuring), and lifestyle (sleep hygiene) components, to individual participant’s responses. Throughout the course, participants are also provided with the opportunity to engage with an optional community that comprises weekly discussions with a sleep expert (live text-based question-and-answer sessions), peer-to-peer discussion forums, and personal message walls. Comments on the Sleepio community can be rated by other users according to helpfulness, and color coding is used to signpost the most helpful users. On completion of the first 6 core sessions with The Prof, users become “graduates” and gain access to a graduate-only discussion forum. Active and consistently helpful graduates are identified by an external moderator and invited to volunteer to become “greeters,” which means they are able to send a welcome greeting to new users when they start Sleepio.

Several factors have been associated with greater levels of engagement with and lower rates of attrition from Web-based interventions, including interactivity, tailored content, social networking, and reminders [22-24]. However, there has been little evaluation of the role of online communities that are embedded within specific intervention programs, such as the Sleepio community. Understanding the user experience of such communities may provide useful insights for researchers, clinicians, and developers keen to maximize engagement with Web-based programs and reduce dropout.

The aim of this study was to explore, using a qualitative methodology, the experiences of individuals undertaking the Sleepio intervention who chose to engage with the optional online community. In particular, the study aimed to elicit the reasons for deciding to engage with the community, as well as any perceived benefits and disadvantages.

## Methods

### Procedure

We developed an online survey (see Textbox 1 for questions) using Bristol Online Surveys (University of Bristol, Bristol, UK). Next, we posted an invitation to the community discussion forum inviting users to participate. In addition, we sent an email invitation to 970 individuals who had previously or were currently working through the Sleepio program to participate in this study. Included within each invitation (and reminder) was a link to the study website, which explained the nature of the research and that ethical approval had been obtained from the University of Nottingham, Nottingham, UK. Each user, after indicating their consent, also provided their Sleepio username and date of birth in order to link their usage of the program with the responses they provided in this study.

**Interview questions on an online survey regarding usage of the Sleepio digital sleep intervention.**

1. The Sleepio community is an optional part of the program; why did you decide to access it?
2. What are the benefits to you from accessing the community?
3. What do you value most about the Sleepio community?
4. How does it help? Could you give some examples of how it has helped you?
5. Can you describe any disadvantages? If so, what are they and do you have an idea how they can be resolved?

### Data Analysis

We analyzed the responses to each question according to the principles of inductive thematic analysis, as described by Braun and Clarke [25]. Each set of responses was read multiple times by 2 authors (NC and RS) in order to gain familiarity with the data and to identify potential emerging themes. Next, these 2 authors discussed their preliminary analysis and together created a thematic framework that was then used to analyze all the responses independently. After further discussion, the authors (NC and RS) confirmed a set of themes, and identified and extracted relevant data to represent each of these themes. The remaining authors then reviewed the themes for coherence.

## Results

In total, 100 respondents (70/100, 70% female; mean age 51 years, range 26–82 years) completed the online survey. Most respondents had started Sleepio with chronic sleep problems (59/100, 59% up to 10 years; 35/100, 35% >10 years) and had actively engaged with the online community (85/100, 85%) had made a discussion or wall post). At the time of the survey, respondents had used Sleepio for a median of 12 weeks (range from 3 weeks to 2 years).

### Reasons for Engagement With the Sleepio Online Community

Respondents described 5 key motivations that led them to engage with the online community: (1) connecting with people facing similar issues, (2) seeking personalized advice, (3) curiosity, (4) being invited by other members, and (5) wanting to use all available sleep improvement tools.

#### Connecting With People Facing Similar Issues

For many, the motivation to engage with the online community stemmed from a need to connect with others facing similar issues. Users described how they wanted to compare their experiences of sleep disturbance with others’, and the community would provide a platform to do this. For many, the need to connect with others who “understand” what they were going through was of critical importance. For example, one user stated, “It was important for me to connect with the community as they were the people who were actually going through the program with me” (female, 60 years).

#### Seeking Personalized Advice

For some, the decision to access the online community arose from the need to ask a question, clarify how to do something, or use it as an additional source of information to supplement what they had learned during the CBT sessions. This helped individuals to obtain as much information as possible to help them overcome their sleep problems, provided them with advice on the practical aspects of implementing what they had learned during the course, and clarified what may or may not work. In the words of one participant, it allowed her “to ask detailed questions about how to implement certain aspects of the Sleepio course, and to ask advice about techniques” (female, 53 years).

#### Curiosity

For some users, curiosity was their motivation to access the online community. In particular, some described having no previous experience of online communities, discussion forums, or social media. As a consequence, they were interested to learn more about the Sleepio community, how it works, and the content of messages posted by other users. After having browsed the discussion forum to determine its potential relevance, most recognized that it was a potentially valuable and integral part of the Sleepio course: “It was there and I was curious so went for a look, and immediately realized how potentially valuable it was to me” (female, 43 years).

#### Being Invited by Other Members

A small number of users mentioned receiving an email from a graduate greeter inviting them to join the online community. While some had initially thought these invitations were automatically generated, on realizing it was indeed a real person they accepted the invitation. For others, there was no initial uncertainty and the invitation motivated them to access the community: “I received a message off someone and decided to have a look” (female, 32 years).

#### Wanting to Use All Available Sleep Improvement Tools

Some saw the online community as an integral part of the treatment program. As a result, users described their willingness to engage with the community in order to maximize their chances of sleep improvement: “It was part of the program offered and it was so important to me to try and improve my sleeping pattern I wanted to access all that was offered to give myself the best opportunity to achieve my goals” (no gender given, 56 years).

### Perceived Benefits of Engagement With the Sleepio Online Community

The benefits derived from engaging with the online community were captured in 7 themes: continuous access, reduced sense of isolation, being part of a supportive and nonjudgmental community, personalized guidance and reassurance, positive comparison with others, encouragement to keep going, and altruism.

#### Continuous Access

The availability of the online community, 24 hours per day and 7 days per week, was appreciated, with many users describing how they benefited by simply knowing the community was accessible whenever they needed it. Beyond this, users could access the community and engage with it, “in my own time” (female, 45 years), with some describing how this was very helpful when engaging with sleep restriction tasks (ie, one of the tasks within the therapy part of the program), for example, “fill some of the hours when I have to stay awake” (female, 63 years). Moreover, easy access to the community meant that users could quickly solicit information or advice: “The immediacy of being able to connect and get answers” (female, 61 years).

#### Reduced Sense of Isolation

On accessing the online community, one of the most immediate benefits that users described was realizing that they were not the only person to be experiencing sleep-related problems: “It made me realize the extent to which sleep deprivation is affecting so many people and that you are not unique” (female, 64 years). Furthermore, the knowledge that they were no longer alone appeared to be of comfort for several: “Knowing you are not alone in your experiences means a lot” (female, 56 years). This was particularly noted when they felt isolated and without an adequate support network: “Sleep problems and insomnia is the loneliest place in the world” (female, 44 years).

#### Being Part of a Supportive and Nonjudgmental Community

The online community was considered a safe venue through which users could obtain much needed support: “It’s a safe place to talk about sleeping problems without them being dismissed as trivial” (female, 41 years). Indeed, users described how they felt that they could ask a question, discuss a personal issue, or simply vent without fear of ridicule or judgment. As a result, engaging with the online community appeared to be as important as the actual program itself in terms of supporting their efforts to improve their sleep. Several noted the “willingness of the community to engage, especially with newcomers to the program” (female, 68 years), and how their engagement with a community of “respect,” “understanding,” and “empathy” was in itself a great comfort.

#### Personalized Guidance and Reassurance

The Sleepio community appeared to be an important source of information and advice, and many users described examples of specific questions they had, that were then answered by other users: “It gave me answers that I needed to know and how to handle different situations, such as when being away on a trip” (female, 68 years). This was particularly evident during the early stages of the course, where many users described initial concerns or uncertainties: “Helped when I didn’t understand the format especially during the first couple of weeks” (female, 56 years).

#### Positive Comparisons With Others

By reading the stories, experiences, and updates posted by others, users were able to directly compare their own progress with that of others. In so doing, users described 2 distinct types of comparison. First, some users described how they compared themselves with others who were doing better, and that this was a source of inspiration: “seeing that others have succeeded” (female, 32 years). Second, and conversely, some described how they felt better after having read that others were in a much worse position than they were: “I...realized that there were many people with much worse issues” (male, 55 years).

#### Encouragement to Keep Going

Several users described how engagement with the online community motivated them to keep going with the course. This was particularly evident during challenging aspects of the course, such as the sleep restriction task: “I value being able to read other people’s experiences, how they have coped, especially when some weeks are challenging, such as the sleep restriction part of the program. I value feedback to my comments because they have been positive and motivating, it encourages me to persevere” (female, 56 years).

#### Altruism

For several users, it appeared important to be able to provide information, advice, and support, as well as to receive it. In so doing, these users clearly wished to give something back to the community: “The opportunity to help others which I like doing, is satisfying and enables me to return some of the benefit I have gained” (female, 77 years). For some, the act of giving back also helped them address their own sleep problems: “I’ve also found that helping others gives me insights into my own sleep problems” (male, 67 years).

### Perceived Disadvantages of Engagement With the Sleepio Online Community

The disadvantages of using the online community focused on 5 areas of concern: design and navigation issues, uncertain quality of user-generated content, negative comparisons with others, excessive time commitments, and data privacy concerns.

#### Design and Navigation Issues

Some community users felt that some aspects of the onscreen user interface could be improved and described problems they had experienced while using it. Specifically, some found the interface difficult to navigate, especially if they did not have any prior experience of using forums or other social media: “I’ve never use a community chat before so was a bit nervous and unsure of how it worked” (female, 45 years). Some felt that navigating their way through conversations was difficult: “I often found the discussions hard to follow as it was sometimes difficult to see which questions were being answered and to follow a sequence of questions and answers through when other questions and answers popped up in the middle” (female, 68 years). Additionally, it wasn’t always clear to users where to post messages or how to search for information that was relevant to their particular problem: “Maybe a better search function would be a great way to find the posts that are relevant to my problem” (male, 68 years).

#### Uncertain Quality of User-Generated Content

Within the community, a vast amount of information and advice was shared online and some users appeared critical of the scientific evidence underpinning user-generated content: “Sometimes people post suggestions (about diet, natural remedies...) that are not supported by scientific evidence which I find confusing” (female, 38 years). This disadvantage was rooted in the fact that “the community aren’t sleep research experts” (male, 39 years) and, while often the information or advice may be based on experience, “their information is not reliable and you don’t know who they are, to be able to trust what they say” (female, 57 years). This issue was considered especially problematic if users were posting to help someone clearly in distress: “So the possibility of ill advice for someone who needs a doctor was the only possible downside I saw, not for me, but for another member” (female, 58 years).

#### Negative Comparisons With Others

For some users, reading about other people’s experiences was on occasion problematic. In some instances, reading about the stress, anxiety, and distress being experienced by others was in itself a source of anxiety and stress: “I saw how others struggled, found it too tough, and gave up: this just scared me, made me anxious, tense and probably worked against what the program is meant to be doing” (female, 29 years). However, some users described similar feelings when others posted about their success: “Reading how wonderful people feel now they are improving after a few days of the program!!!, Not so nice to see this if it isn’t working for you after a few weeks longer” (female, 59 years).

#### Excessive Time Commitments

Engaging with the online community was time consuming, and some users described feeling “overwhelmed” by the volume of messages being posted. As a result, some felt they were spending too much time in the community: “I can find myself spending way too much time or trying to make the time to see all” (female, 59 years). For others, reading messages was “addictive” and something that could “eat up time unnecessarily.” However, time spent was meaningful, as users undertook to offer advice and support through posting messages or replying to questions: “It takes a lot of time to compose a meaningful helpful message, particularly to someone who is struggling” (female, 61 years).

#### Data Privacy Concerns

A small number of users expressed concerns regarding the perceived privacy of the discussion forum. In particular, some felt it was not sufficiently clear that it was openly accessible, and therefore any message posted to the forum could, in theory, be read by anyone. Additional concerns were focused on the belief that user-generated content could be accessed through Internet search engines. As a consequence, some users described feeling upset and explained how this had led them to disengage from making an active contribution (ie, posting) or to be more discerning about the content they uploaded to the forum: “Even though the community is anonymous and members’ names are not public, the content of the messages is public and that was quite upsetting to discover that... I have been more careful since discovering that in terms of what I post” (female, 61 years).

## Discussion

The problem of attrition within digital interventions has been widely documented [26]. Despite this, there has been little work exploring how online communities can support long-term engagement with Web-based therapy. In this study, we wished to explore Sleepio program participants’ decisions to engage with and their experiences in the optional community. What is evident from their responses is that their reasons for engaging with the community varied but that their overall experiences were broadly positive and appeared to contribute to their sustained engagement with the Sleepio program and, for some, sleep improvement.

Arguably, one of the most important features of the community is that it is available at any point of the day or night, 7 days a week, and regardless of time zone. Furthermore, it is convenient and provides an immediate source of support to individuals who, according to their own stories, may feel isolated, exhausted, frustrated, and despondent as a result of their sleep problems [27].

Our results also revealed that the Sleepio program was considered demanding, especially the sleep restriction component. Previous work has indeed identified important challenges in the delivery of sleep restriction therapy to patients with insomnia [28,29], particularly in relation to adverse events and likely implementation or adherence challenges. Therefore, as an adjunct tool for individuals engaging with Web-based therapy for insomnia, the presence of a community, and in particular an asynchronous discussion forum, may be particularly helpful in terms of providing support.

For many, the community offered a means to connect with other people who not only had sleep problems but also were undertaking the Sleepio program. Indeed, this notion of connection was also reflected in responses concerning perceived benefits of the community. These findings resonate with previous research that has demonstrated how online communities can act as important venues through which individuals facing challenging health problems can soon feel less isolated and more supported [30-32], regardless of how much they actively engage with the community. Indeed, evidence suggests that community users typically vary in their level of engagement, ranging from those who post messages regularly through to those who simply lurk (ie, read messages only), but their experience may be equally rewarding [33].

The Sleepio community was certainly an important source of information, advice, and ideas for users, and this is also consistent with previous literature [30]. In particular, users sought and received a high volume of experiential information, which appeared to be well received by others, not least because it was understandable, relevant, and credible. Previous research has found information provision to be one of the key functions within online support communities [34,35]. We found a range of information being exchanged, including factual information, advice, and personal suggestions for coping strategies and management. Going forward, it would be useful to undertake a more fine-grained analysis of the specific types of informational support requested and exchanged within such an online community and how this relates to engagement with the various components of the Sleepio program.

In addition to the aforementioned examples of how the community benefited users, we also identified a clear motivational function. It was evident from responses that participation in the community provided the impetus to keep going, and users offered messages of support and encouragement, especially during difficult parts of the program (eg, sleep restriction). Indeed, previous research has identified poor adherence to CBT interventions for insomnia, particularly in the context of implementing behavioral advice [36]. Our results suggest that engagement with the online community may be one avenue through which user motivation and adherence can be supported. Such findings are comparatively novel within the broader literature, since the majority of online communities are either standalone or embedded within a program, but such online communities embedded within a program have typically not been evaluated in isolation. In future, researchers may wish to examine more explicitly the means through which engagement with online communities motivates individuals to persist with Web-based therapy for insomnia; however, our findings present some useful starting points for investigation (eg, provision of information and advice, and social comparison).

Our analysis also revealed some disadvantages arising from the use of the online community. While some of these (ie, design and navigation issues, and privacy concerns) were clearly associated with operational aspects of the Sleepio community and therefore can be easily addressed, others have been reported elsewhere in the literature. For example, some users were concerned about the accuracy of information posted by other members of the community. However, our analysis did not consider the accuracy of any information exchanged between users. In the future, it would be beneficial to ascertain the extent to which user-posted information may be inaccurate, not applicable, or perhaps unsafe. Despite these concerns, other studies that have examined the accuracy of information posted by users of discussion forums suggest that this is not a common problem but may only be more problematic in communities with low levels of activity [37]. Indeed, other users swiftly correct any inaccurate information that is posted [38]. The Sleepio community, although not continuously monitored by staff, has several mechanisms in place to create a safe environment. First, posts can be flagged by other users when considered not in line with the terms of use. Second, keywords that, for example, display a medical risk or personal information, are flagged automatically. In addition, expert sessions take place on a weekly basis to provide support by a clinical psychologist.

While we found that reading about the experiences of other users could be helpful, we also found that, for some, this actually had a negative impact on their well-being. In particular, some considered reading about the “horror stories” posted by others to be unhelpful, upsetting, and distressing. Such findings have also been reported elsewhere in the literature and may reflect the fact that many individuals use discussion forums to vent [32] or offload their daily hassles and bad experiences [39]. However, for those who read such messages, the impact can be problematic. While this is a challenging issue to address, one solution may be in the design and organization of forums, where subforums may be used to channel different types of user-generated content into specific locations, for example, sleep problems while being at college, or success stories. Similar mechanisms are seen in face-to-face versions of group therapy; while groups are helpful, they can sometimes be experienced as negative.

One final challenge facing users was the amount of time it took to engage with the online community and in particular all the discussions taking place. This issue was further exacerbated when users wished to provide help and support to others, as they viewed composing a helpful and tailored message to be time consuming. As online communities, such as this, continue to flourish, this issue is likely to persist and may indeed worsen. Restructuring forums into subforums may help to address this issue as well, such that users can choose which content they wish to engage with each time they access the community.

### Study Limitations

We acknowledge some limitations to this study. First, despite our best efforts to recruit participants, the sample size is modest, and therefore the extent to which the views expressed by respondents represents the entire pool of Sleepio users is debatable. One obvious concern may be that only those who held particularly positive or negative views toward the community chose to participate in this study. Similarly, since the majority of our respondents were active users of the community, future research needs to consider the experiences of those who either engaged in a limited way or not at all. Second, the data for this study were captured by a single online survey, and it could be argued that experiences, views, and attitudes toward the Sleepio community may change over time, as users move through the program. While this is arguably true, we did note a broad range of users (ie, newcomers, users enrolled on the course, and graduates of the course), as well as evidence of respondents recalling specific good or bad experiences within the community in the past. All that said, it would be useful to engage in more longitudinal work to follow the experiences of those engaging with the Sleepio program from the point of entry and to consider how participation in the community contributes to relevant outcomes and indicators of adherence to the Sleepio intervention content. Third, since the focus of this study was on users’ experiences, we know little about those who chose not to engage with the community and their reasons behind this decision.

### Conclusion

Despite some concerns, members regarded the Sleepio community as a valuable resource. Online communities may be a useful means through which to support long-term engagement with Web-based therapy for insomnia.

## Abbreviations

CBT: cognitive behavioral therapy
